# Multi-Scale Modeling and Experimental Validation of Thermo-Mechanical Responses in Femtosecond Laser Micromachining of Copper

**DOI:** 10.3390/ma19071391

**Published:** 2026-03-31

**Authors:** Jianguo Zhao, Xu Han, Fang Dong, Sheng Liu

**Affiliations:** 1 The Institute of Technological Sciences, Wuhan University, Wuhan 430072, China; jianguozhao_zjg@whu.edu.cn (J.Z.); leo_han@whu.edu.cn (X.H.); 2 School of Power and Mechanical Engineering, Wuhan University, Wuhan 430072, China

**Keywords:** laser ablation, multiscale simulation, TTM-FEM, TTM-MD

## Abstract

Femtosecond laser micromachining is a cornerstone of high-precision manufacturing, yet its multi-scale dynamics require a self-consistent bridging from atomic transitions to macroscopic morphology. This study establishes a multi-scale framework where Density Functional Theory (DFT) calculates temperature-dependent electronic thermal properties to inform both Two-Temperature Model-Molecular Dynamics (TTM-MD) and Finite Element Method (TTM-FEM) simulations. By comparing atomistic and macroscopic results, we systematically investigate the thermal-mechanical responses of copper ablation. The macroscopic TTM-FEM model, employing a removal criterion based on the enthalpy of vaporization, achieves high predictive accuracy for ablation depths in the low-to-medium power range up to 300 mW. However, a significant divergence at higher powers (>400 mW) highlights the physical transition from surface evaporation to phase explosion. Concurrently, the TTM-MD simulations provide microscopic insights into the transient temperature and stress evolution, establishing a physically synchronized link between atomic-scale dynamics and macroscopic results. This work defines the applicability boundaries of evaporation-based macroscopic models and provides a validated predictive tool for optimizing laser processing parameters in precision engineering.

## 1. Introduction

The unique advantages of femtosecond lasers, characterized by their ultrashort pulses and extremely high peak power, have revealed broad application prospects in frontier fields such as aerospace, microelectronics, and biomedicine [1,2,3,4,5]. Their distinctive cold processing capability minimizes the heat-affected zone, thereby enabling ultra-fine processing of materials [6,7,8,9]. However, the interaction between a femtosecond laser and matter occurs at extreme spatio-temporal scales and involves highly complex physical phenomena [10,11,12,13], making it difficult to deeply investigate the underlying mechanisms through purely experimental means [14,15,16,17]. Consequently, the development of high-fidelity numerical simulation methods has become an indispensable tool for revealing ablation mechanisms and achieving precise process control [18,19].

Currently, accurate numerical simulation faces the severe challenge of the multiscale discrepancy [20,21,22,23]. The femtosecond laser ablation process exhibits significant multi-scale characteristics: temporally, it spans from electron excitation (femtoseconds) to lattice heating (picoseconds) and finally to material ejection (nanoseconds); spatially, it covers multiple orders of magnitude from the electron absorption depth (nanometers) to the macroscopic ablation crater (micrometers) [24,25,26]. This inherent cross-scale nature renders single-scale simulation methods inadequate [27]. While macroscopic continuum models are computationally efficient, they cannot capture atomic-scale ultrafast phase transition dynamics [28]. Conversely, although microscopic Molecular Dynamics (MD) models can accurately describe atomic interactions, their immense computational cost prevents them from directly predicting the final macroscopic ablation results [29,30].

At the microscopic scale, the Molecular Dynamics coupled with the Two-Temperature Model (TTM-MD) simulations have played a crucial role. For instance, Ji et al. [31] improved the accuracy of model parameters through first-principles calculations, while Zhou et al. [32] focused on optimizing algorithms to simulate larger atomic systems. Such atomistic-to-continuum models have achieved great success in revealing non-equilibrium thermodynamics and stress wave evolution. Lian et al. [33] investigated the influence of initial temperature on the ultrafast thermomechanical response of aluminum thin films under femtosecond laser irradiation by employing molecular dynamics coupled with the two-temperature model (MD-TTM) and pump-probe ultrafast imaging techniques. Ganesan et al. [34] employed a hybrid two-temperature model and molecular dynamics (TTM-MD) simulation approach to investigate the influence of crystallographic orientation and microstructural configurations on the ultrafast thermomechanical responses and phase transition processes of gold thin films under femtosecond laser irradiation. Despite the self-consistent coupling in the M-TTM-MD framework [35], the extreme computational cost of MD limits its application to sub-micron and picosecond scales. This inherent spatial-temporal constraint creates a fundamental ‘validation gap,’ as it precludes a direct, quantitative correlation with macroscopic experimental observables, such as final ablation depth and wide-area surface morphology [36].

At the macroscopic scale, while Finite Element Method (FEM) models can bridge the gap to experimental dimensions, they often suffer from mechanistic incompleteness due to a deficiency in microscopic physical insights and imperfect multi-physics coupling. An accurate simulation requires a framework capable of simultaneously handling extreme non-equilibrium thermodynamics and dynamic mechanical responses; however, current research frequently fails to address both aspects adequately. For example, although Omeñaca et al. [37] and Dong Liu et al. [38] accurately described the thermal processes using the TTM, the lack of a solid mechanics module prevented them from capturing the associated mechanical responses and the resulting structural integrity of the target. Other works have focused more on mechanical behavior, such as the model by Akarapu et al. [39], which included thermal stress and material failure, it is based on a single-temperature equation for long-pulse lasers and cannot capture the characteristics of stress evolution under the action of femtosecond lasers. Even models that consider both the TTM and solid mechanics, such as the research by Tsibidis et al. [40], are often limited to plastic deformation under sub-melting conditions and do not involve actual material ablation and removal. The work of Redka et al. [41] enhanced FEM accuracy by integrating an equation of state to capture complex ablation dynamics. However, its 1D nature precludes modeling 3D surface morphologies, while the inherent nonlinear complexity increases computational costs, limiting its utility for exploring novel phenomena under extreme conditions. While Liu et al. [42] successfully captured the phase-change boundary evolution using FEM, the model is limited to thermal transport and neglects the mechanical response, such as thermo-elastic stress and material fracturing, which are critical in brittle stone fragmentation. To bridge the gap between simulation efficiency and physical accuracy, Xu et al. [43] implemented a 2D axisymmetric TTM framework that accounts for ballistic electron effects via an optimized effective optical penetration depth. While this approach significantly improves the prediction of ablation depth and width for copper thin films, it simplifies the ablation process as a purely thermal-phase transition, overlooking the complex thermo-mechanical stress fields inherent in ultrashort pulse interactions. Consequently, a pervasive gap exists in the academic community for a comprehensive multi-physics framework capable of fully coupling three core physical domains: the two-temperature model, solid mechanics, and dynamic ablation deformation. Such an integrated model not only elucidates the co-evolution of thermal and mechanical responses but also provides an intuitive visualization of the material ablation process and its final morphological features.

In light of these limitations, multiscale modeling has emerged as a promising paradigm to reconcile microscopic physical fidelity with macroscopic computational efficiency. Researchers have sought to integrate the advantages of various scales through concurrent coupling or hierarchical information passing methods, aiming to construct a comprehensive picture of the ablation process. Studies have successfully coupled macroscopic FEM with atomic-scale Kinetic Monte Carlo to achieve cross-scale coupling (Calogero et al. [44]), this framework is ill-suited due to its focus on near-equilibrium thermal melting. Similarly, multiscale models have been developed for other material systems, such as the work by Zhao et al. [45] on semiconductors; however, these models cannot be directly transferred, since the underlying ablation physics in metals under such extreme non-equilibrium states is fundamentally distinct.

While multi-scale and multi-physics challenges have been explored individually, a comprehensive framework capable of bridging the scale gap through unified physical inputs remains a necessity. Such a framework is vital for elucidating the non-equilibrium co-evolution of thermal-mechanical responses, thereby enabling the precise prediction of macroscopic ablation morphology and residual material states across diverse fluences. However, research on such integrated multi-scale models remains in its infancy, and filling this critical gap is the primary objective of the present work.

To address these challenges, we propose an experimentally validated, integrated multi-scale framework for the femtosecond laser micromachining of copper. In this approach, temperature-dependent electronic thermal properties, precisely derived from First-principles Density Functional Theory (DFT), serve as the fundamental physical bedrock to independently drive both a microscopic TTM-MD model and a macroscopic TTM-FEM model. This unified dual-track strategy ensures intrinsic physical consistency, enabling a direct cross-scale correlation between atomistic thermo-mechanical dynamics and macroscopic responses. Unlike traditional single-scale studies, our framework systematically evaluates the predictive boundaries of the continuum model by benchmarking it against surface evaporation kinetics and microscopic insights. It is demonstrated that while the evaporation-based criterion maintains high fidelity in the low-to-medium power regime (up to 300 mW), the divergence at higher powers (>400 mW) is physically elucidated as a regime transition toward phase explosion. The established applicability boundaries provide actionable guidance for optimizing laser parameters in industrial precision manufacturing, ensuring superior surface integrity by strategically avoiding chaotic ablation regimes.

## 2. Materials and Methods

### 2.1. Experimental Setup and Characterization

In this section, the experimental platform and characterization techniques used to validate the multi-scale model are described. The setup focuses on capturing the surface morphology and ablation depth of copper targets under varying femtosecond laser irradiation conditions. To provide a clear overview of the experimental conditions, the key technical specifications of the laser system and the high-resolution characterization instruments are summarized in Table 1.

**Figure 1:**
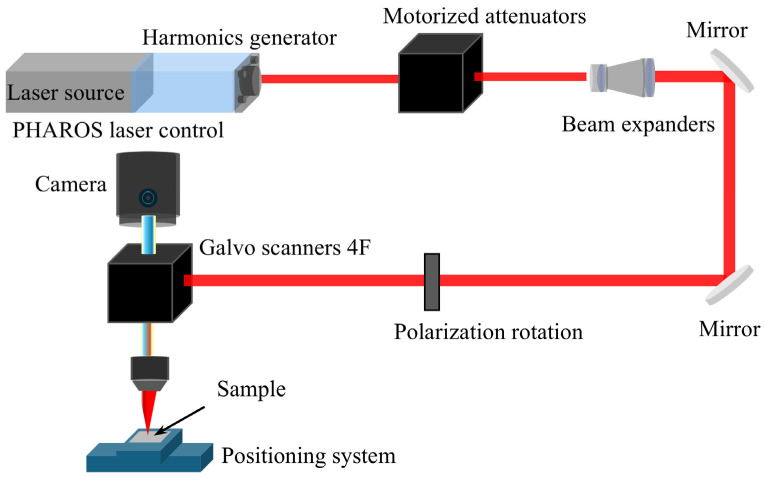
Schematic of the femtosecond laser ablation system.

The experimental configuration of the femtosecond laser micromachining system is illustrated in Figure 1. Single-pulse ablation was performed on bulk copper samples (99.99% purity, Haochen Metal Materials Business, Hefei, Anhui, China). To strictly ensure single-pulse ablation conditions, the pulse displacement (*d* = *v*/*f*, where *v* is scanning speed and *f* is repetition rate) was maintained significantly larger than the beam diameter (2*w*_0_). This configuration eliminates cumulative thermal or incubation effects, ensuring each crater reflects the intrinsic physical response of a standalone laser-material interaction on an undisturbed surface. To ensure a consistent surface response, the targets were prepared by a multi-stage polishing process using silicon carbide (SiC) abrasives and subsequent SiC-based suspensions. This treatment produced a mirror-like finish with an estimated surface roughness (*R*_a_) of less than 20 nm.

The laser source was a PHAROS [46] femtosecond laser system (Light Conversion, Vilnius, Lithuania) [47] operating at a central wavelength of 1028 nm with a pulse duration of 190 fs. The beam energy was controlled by a precision attenuator. To achieve micro-scale precision, the beam was focused onto the sample using a 50× high-numerical-aperture objective (NA = 0.42; M Plan Apo 50×, Mitutoyo, Kawasaki, Japan), where the beam waist radius (*w*_0_) used for all calculations was determined to be 0.9 μm based on the theoretical diffraction limit *w*_0_ = *M*^2^/(πNA). This value is strongly supported by experimental observations (detailed in Section 3.1), where measured ablation crater diameters at low fluences are consistently around 2 μm, showing excellent agreement with the calculated beam waist radius. While average power (*P*_avg_) was utilized as the primary monitoring variable during experiments for high-precision, real-time feedback, all values were converted to absorbed fluence (*F*) using *F* = (1 − *R*) *P*_avg_/(*f* π*w*_0_^2^) to maintain consistency in the physical mechanism analysis. This conversion ensures the accuracy of the energy density input in our multi-scale models and facilitates a direct correlation between the experimental parameters and the intrinsic thermomechanical responses.

We systematically investigated the ablation morphology by varying the average laser power from 100 mW to 600 mW. After irradiation, the surface morphologies of the craters were observed using Field Emission Scanning Electron Microscopy (FE-SEM, TESCAN MIRA3 LMH, Tescan Orsay Holding, a.s., Brno, Czech Republic) [48]. The corresponding 3D topographical maps and ablation depths were characterized via 3D Optical Profilometry (WLI, Zygo NewView 9000, Zygo Corporation, Middlefield, CT, USA) [49]. For each crater, the ablation depth was defined as the vertical distance from the unperturbed pristine surface (defined as the 0 μm reference plane) to the lowest point of the crater floor (maximum depth). To ensure statistical reliability, the final depth for each power setting was determined by averaging the maximum depths measured from five independent craters. Furthermore, the local strain distributions were analyzed by Electron Backscatter Diffraction (EBSD) using a high-resolution FE-SEM (TESCAN CLARA, Tescan Orsay Holding, a.s., Brno, Czech Republic) [50] equipped with an EBSD detector. These integrated experimental results serve as the primary benchmark for validating the predictive accuracy of the subsequent multi-scale simulations.

### 2.2. Overview of the Framework

**Figure 2:**
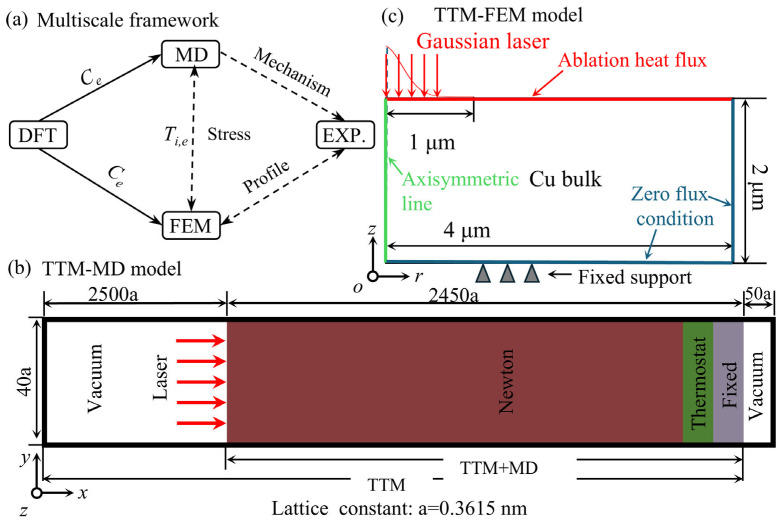
Multiscale simulation framework for femtosecond laser ablation. (**a**) Flowchart of the framework. (**b**) Schematic of the TTM-MD model. (**c**) Schematic of the TTM-FEM model. The triangles denote the fixed supports representing the mechanical boundary conditions.

The multi-scale simulation framework developed in this study integrates DFT, TTM-MD, and TTM-FEM to bridge the vast spatio-temporal gap between microscopic electron-lattice dynamics and macroscopic morphological evolution, as illustrated in Figure 2. Unlike traditional sequential coupling methods, our approach utilizes temperature-dependent electronic thermal properties, precisely derived from DFT calculations [51], as a consistent physical input for both the TTM-MD and TTM-FEM governing equations. This ensures that the non-equilibrium thermodynamic descriptions at both atomic and continuum scales are physically synchronized. Within this framework, the microscopic TTM-MD model (Figure 2b) captures ultrafast atomic-scale responses ranging from void nucleation and transient stress wave propagation to phase transition dynamics, providing a physical benchmark for the stress-driven material removal mechanism. Simultaneously, the macroscopic TTM-FEM model (Figure 2c) is implemented to predict the final ablation crater morphology by coupling the two-temperature equations with solid mechanics. Material removal in the FEM model is handled via the deformed geometry technique (ALE) based on a surface evaporation criterion. The validity of the framework is established by directly comparing the transient temperature and stress evolution between MD and FEM, while the final predicted crater depths from the FEM model are validated against experimental results.

### 2.3. Computational and Modeling Details

To provide accurate thermodynamic parameters for the multi-scale framework, Density Functional Theory (DFT) calculations were performed using the Quantum Espresso package [52] (Quantum ESPRESSO Foundation, Scuola Internazionale Superiore di Studi Avanzati, Trieste, Italy) with the PBE exchange-correlation functional [53]. The computations utilized a copper primitive cell with a kinetic energy cutoff of 100 Ry and an SCF convergence threshold of 10^−9^ Ry. An 11 × 11 × 11 Monkhorst-Pack k-point grid was employed to ensure Brillouin zone sampling accuracy. These calculations yielded the temperature-dependent electronic heat capacity (*C*_e_), which is physically defined as *C*_e_(*T*_e_) = *T*_e_(*∂S*_e_/*∂T*_e_) [54]. In this study, *C*_e_ was numerically evaluated using the finite difference method based on the calculated electronic entropy (*S*_e_):

(1) $$C_{e}\left(T_{e}\right)\approx T_{e}\frac{S_{e}\left(T_{e}+\Delta T_{e}\right)-S_{e}\left(T_{e}-\Delta T_{e}\right)}{2\Delta T_{e}}$$

where *T*_e_ represents the temperature increment between discrete DFT data points. This *C*_e_ was subsequently incorporated into both the TTM-MD and TTM-FEM governing equations to account for the extreme non-equilibrium state during laser irradiation. To accurately incorporate the temperature-dependent electronic specific heat *C*_e_ into the multi-scale model, a mathematical fitting was performed based on theoretical data. The relationship between *C*_e_ and the electronic temperature *T*_e_ is expressed by a combination of a constant term and a high-order polynomial modulated by a Gaussian-like exponential decay function [55]:

(2) $$\;C_{e}=C_{0}+\left(a_{0}+a_{1}X+a_{2}X^{2}+a_{3}X^{3}+a_{4}X^{4}\right)\exp\left(-{\left(AX\right)}^{2}\right)$$

where *X* = *T*_e_/1000 represents the scaled electronic temperature.

Large-scale TTM-MD simulations were conducted using the LAMMPS [56] package (Large-scale Atomic/Molecular Massively Parallel Simulator, Sandia National Laboratories, Albuquerque, NM, USA) with the “fix ttm/mod” [57,58] implementation to account for laser-induced heating. The evolution of the electronic temperature *T_e_* is described by the continuum heat diffusion equation:

(3) $$C_{e}\rho_{e}\frac{\partial T_{e}}{\partial t}=\nabla \left(\kappa_{e}\nabla T_{e}\right)-G\left(T_{e}-T_{a}\right)+S(x,t)$$

where *C*_e_, *ρ*_e_, and *κ*_e_ represent the electronic specific heat, density, and thermal conductivity, respectively. The heat exchange between the electrons and the atomic subsystem occurs via an inhomogeneous Langevin thermostat, with *G* defining the coupling strength. The specific heat *C*_e_ depends on *T*_e_ according to a high-order polynomial modulated by Equation (3), ensuring consistency with our DFT-calculated electronic properties.

The simulation was performed on a system consisting of approximately 15.68 million atoms. The simulation box, with dimensions of 40a × 40a × 5000a (a = 0.3615 nm), was partitioned into several functional regions. Periodic boundary conditions (PBCs) were applied in the lateral directions (*y* and *z* axes) to eliminate unphysical boundary effects from the simulation box side-walls, ensuring that atomic motion and force states were not disturbed by artificial lateral constraints. Along the laser incident direction (*x*-axis), a sufficiently large vacuum layer was placed to allow the free expansion of the laser-induced ablation plume, while thermostat and fixed layers were implemented at the bottom to mimic the heat dissipation and mechanical behavior of a semi-infinite substrate. While the experimental laser beam follows a Gaussian distribution, the MD simulation represents a localized region extracted from the beam center. Given that the lateral dimension of the MD box (~14.5 nm) is nearly three orders of magnitude smaller than the beam waist radius (*w*_0_ = 0.9 μm), the spatial gradient of laser intensity within this microscopic area is negligible.

After initial equilibration at 300 K, the ablation process was simulated in an NVE ensemble for 200 ps with a time step of 1 fs. The laser energy was deposited following the Beer-Lambert law. Structural evolution, including phase transitions and void nucleation, was analyzed via OVITO [59] (Open Visualization Tool, OVITO GmbH, Darmstadt, Germany) using Common Neighbor Analysis (CNA) [60] and the Centrosymmetry Parameter (CSP) [61] to distinguish between crystalline, liquid, and disordered local environments. The TTM-MD parameters used in the model can be found in detail in Table 2.

These physical parameters in Table 2 collectively define the energy deposition, transport, and relaxation dynamics during ultrafast laser-matter interaction. The reflectivity (*R*) governs the fraction of incident energy coupled into the material, while the absorbed fluence (*F*) serves as the primary driver for the material’s transition from heating to ablation. The spatial profile of energy deposition is established by the optical penetration depth (*δ*) and the ballistic electron transport distance (*δ*_b_) with the electron diffusion coefficient (De)regulating the rate of spatial heat spread within the excited electronic system. Crucially, the electron-phonon coupling constant (*G*) quantifies the energy transfer rate from the electronic subsystem to the lattice, thereby dictating the non-equilibrium phase transition and the resulting ablation morphology.

The macroscopic response was simulated using a 2D axisymmetric TTM-FEM model implemented on a cylindrical domain (4 μm radius, 2 μm height) via COMSOL Multiphysics® software (v6.2, COMSOL AB, Stockholm, Sweden). This model fully couples the Two-Temperature Model (TTM) with solid mechanics to solve for temperature and stress distributions. In cylindrical coordinates, the energy evolution and heat exchange between the electronic and lattice subsystems are governed by:

(4) $$\;\left\{\begin{array}{c} C_{e}\rho_{e}\frac{\partial T_{e}}{\partial t}=\kappa_{e}\left(\frac{\partial^{2}T_{e}}{\partial r^{2}}+\frac{1}{r}\frac{\partial T_{e}}{\partial r}+\frac{\partial^{2}T_{e}}{\partial z^{2}}\right)-G\left(T_{e}-T_{l}\right)+S\left(r,z,t\right)\\ C_{l}\rho_{l}\frac{\partial T_{l}}{\partial t}=\kappa_{l}\left(\frac{\partial^{2}T_{l}}{\partial r^{2}}+\frac{1}{r}\frac{\partial T_{l}}{\partial r}+\frac{\partial^{2}T_{l}}{\partial z^{2}}\right)+G\left(T_{e}-T_{l}\right) \end{array}\right.$$

where *C*, *ρ*, and *κ* represent the specific heat, density, and thermal conductivity for the electron e and lattice l subsystems, respectively. The laser source term *S*(*r*, *z*, *t*) accounts for the Gaussian spatio-temporal distribution and energy absorption, defined as:

(5) $$S\left(r,z,t\right)=\sqrt{\frac{4\ln2}{\pi }}F\frac{1-R}{\tau }\frac{1}{\delta +\delta_{b}}\exp\left(-4\ln2\frac{t^{2}}{\tau^{2}}-\frac{2r^{2}}{w_{0}^{2}}-\frac{z}{\delta +\delta_{b}}\right)$$

where *F* is the laser fluence, *τ* is the pulse duration. To capture the transient mechanical behavior, the equations of motion in cylindrical coordinates are solved to derive the displacement and stress fields:

(6) $$\left\{\begin{array}{c} \rho_{l}\frac{\partial^{2}u_{r}}{\partial t^{2}}=\frac{\partial \sigma_{r}}{\partial r}+\frac{\partial \tau_{rz}}{\partial z}+\frac{1}{r}\left(\sigma_{r}-\sigma_{\theta }\right)\\ \rho_{l}\frac{\partial^{2}u_{z}}{\partial t^{2}}=\frac{\partial \tau_{rz}}{\partial r}+\frac{\partial \sigma_{z}}{\partial z}+\frac{1}{r}\tau_{zr} \end{array}\right.$$

Material removal was implemented through the Arbitrary Lagrangian-Eulerian (ALE) deformed geometry technique. The surface ablation velocity (*v*_mesh_) is governed by the net heat flux (*Φ*_v_) and the enthalpy of vaporization (*L*_v_) [66]:

(7) $$v_{\mathrm{mesh}}=\Phi_{v}/\left(\rho_{l}\cdot L_{v}\right)$$

Boundary conditions were defined to represent semi-infinite bulk conditions, including non-reflecting mechanical boundaries at the side and top, fixed supports at the bottom, and heat loss (convection and radiation) at the free top surface. Table 3 further delineates the intrinsic thermophysical and mechanical properties of the target material.

Lattice density (*ρ*_l_) and conductivity (*κ*_l_) define the thermal response, while energy thresholds for phase transitions are set by *T*_m_, *T*_v_, and *L*_v_. Thermo-mechanical coupling is incorporated via Young’s modulus (*E*) and Poisson’s ratio (ν). The resulting system with 284,807 DOFs was resolved using the MUMPS solver, selected for its robustness in handling non-symmetric matrices and ALE-based mesh distortions. Its distributed memory architecture and symbolic factorization reuse ensure computational efficiency. Numerical reliability was verified at 600 mW; as shown in Figures S1 and S2 (SI), the convergence of ablation depth and stress fields confirms that the 25.0 nm mesh and 1 fs step accurately capture the ultrafast dynamics, with stability inherently guaranteed for lower power levels (100–500 mW).

## 3. Results and Discussion

### 3.1. Experimental Observations of Ablation Morphology

**Figure 3:**
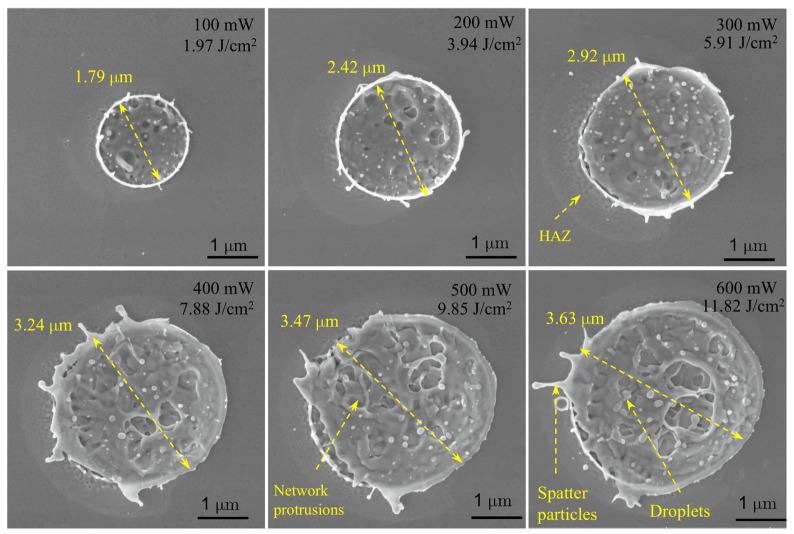
Experimental results of femtosecond laser ablation on copper. SEM images showing the evolution of ablation crater morphology with increasing average laser power (100 mW to 600 mW). Key features such as the Molten rim, HAZ (Heat Affected Zone), Droplets, Network protrusions, and Spatters particles are indicated.

The experimental results demonstrate that the ablation morphology of copper is highly sensitive to the incident laser power, exhibiting a distinct transition in the underlying physical mechanisms, as illustrated in Figure 3.

**Figure 4:**
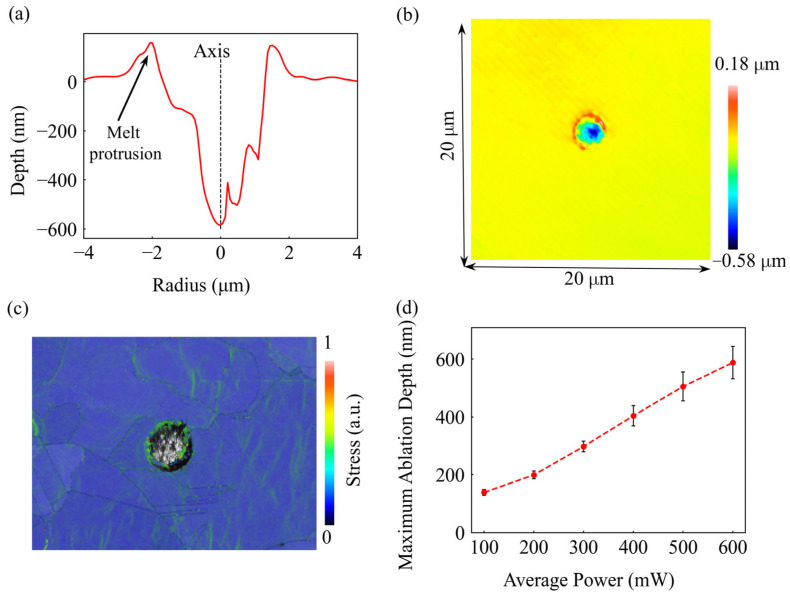
Experimental results of femtosecond laser ablation on copper. (**a**) Radial depth profile of the crater at 600 mW (11.82 J/cm^2^), showing the prominent Melt protrusion at the rim and the central Axis. (**b**) 3D morphology at 600 mW. (**c**) KAM map at 600 mW (11.82 J/cm^2^), (**d**) Maximum ablation depth as a function of average laser power.

Quantitative topographic analysis of the extreme case at 600 mW (Figure 4a,b) reveals a characteristic V-shaped cross-sectional profile, with a maximum ablation depth of approximately 0.58 μm. The prominent melt protrusion at the crater rim confirms the strong coupling between recoil pressure and Marangoni flow during the extreme thermal cycle. Furthermore, the Kernel Average Misorientation (KAM) map at 600 mW (Figure 4c) reveals a high concentration of residual stress at the crater periphery. This mechanical footprint aligns with the intense thermal-mechanical cycles and material redistribution occurring during the ablation process.

Figure 4d presents the quantitative relationship between the average laser power and the measured maximum ablation depth. In the lower power range (100–300 mW), the ablation depth increases steadily and almost linearly with the laser power, aligning with the steady surface evaporation regime, where thermal-mechanical effects contribute to the final material state without dominating the removal depth. However, a significant change in the slope is observed beyond the 300 mW threshold. Between 400 mW and 600 mW, the depth exhibits a more rapid, non-linear growth, which correlates with the transition to the violent phase explosion regime. This quantitative trend further validates 300 mW as the critical boundary between stable material removal and chaotic, explosive ablation, providing a clear benchmark for evaluating the predictive limits of the subsequent numerical models.

### 3.2. Thermal Properties via DFT

**Figure 5:**
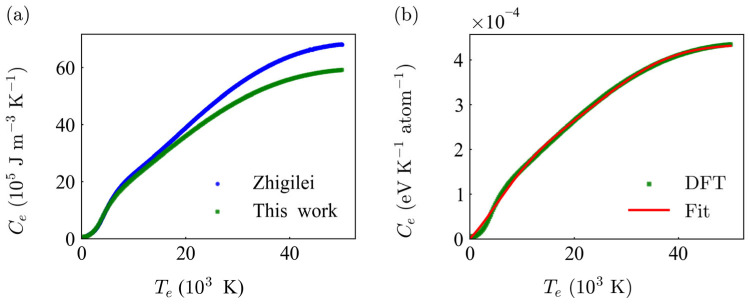
Evaluation and fitting of temperature-dependent electron heat capacity. (**a**) Comparison between the electron heat capacity calculated from Density Functional Theory and the results from the Zhigilei [51] model; (**b**) consistency analysis between the original calculation data and the corresponding numerical fitting results.

The temperature-dependent electronic heat capacity (*C*_e_) of copper, determined via Density Functional Theory (DFT), is presented in Figure 5. These material-specific parameters establish a unified thermodynamic foundation, ensuring that both the microscopic TTM-MD simulations and macroscopic TTM-FEM predictions are physically synchronized and self-consistent.

Figure 5a compares our DFT-calculated *C*_e_ with the widely recognized model by Zhigilei et al. A high degree of consistency is observed between the two curves, particularly in the lower electron temperature range (*T*_e_ < 20 × 10^3^ K). This regime is critical for the initial stages of laser energy absorption and electron-phonon coupling. While a slight divergence emerges at extremely high temperatures (*T*_e_ > 30 × 10^3^ K), the deviation remains within an acceptable range for multi-scale simulations and does not qualitatively alter the predicted thermal-mechanical responses.

To facilitate the integration of these raw data into the Two-Temperature Model (TTM), a numerical fitting was performed. Figure 5b demonstrates the excellent agreement between the original DFT data points and the corresponding fitting curve. By employing these verified, material-specific fitting parameters rather than generic literature values, the subsequent TTM-based models achieve higher robust accuracy in capturing the transient evolution of temperature and stress across different scales.

### 3.3. Microscopic Dynamics via TTM-MD Simulation

**Figure 6:**
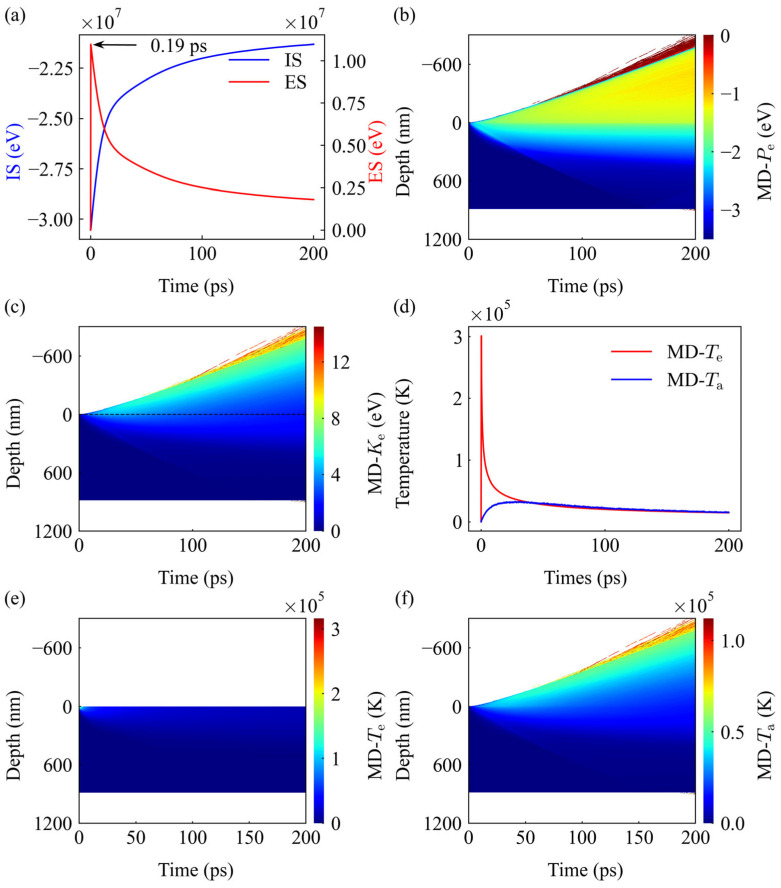
Overview of non-equilibrium dynamics from TTM-MD simulation of copper ablation at 100 mW (1.97 J/cm^2^). (**a**) Temporal evolution of the ion system (IS) and electron system (ES) energies, showing the energy transfer bottleneck. (**b**,**c**) Spatiotemporal evolution of atomic potential energy (MD-*P*_e_) and kinetic energy (MD-*K*_e_). The expansion into the vacuum (Depth < 0) indicates the initiation of plume ejection. (**d**) Evolution of electron temperature (*T*_e_) and lattice temperature (*T*_a_). *T_e_* reaches a peak of 3 × 10^5^ K at 0.2 ps, while *T_a_* thermalizes at approximately 31.5 ps. (**e**,**f**) Spatiotemporal distribution of MD-*T*_e_ and MD-*T*_a_, highlighting rapid electronic heat diffusion preceding lattice thermal expansion.

The TTM-MD simulations provide a comprehensive look into the ultrafast laser-matter interaction, characterized by extreme thermodynamic non-equilibrium. As shown in Figure 6a, a pronounced energy transfer bottleneck is observed between the electronic and ionic systems upon laser deposition. The energy of the electron system (ES) surges instantaneously, peaking at 0.19 ps, while the ion system (IS) responds with a significant delay as electron-phonon coupling progresses. This energy redistribution is visualized in the spatiotemporal maps of atomic potential energy (MD-*P*_e_) and kinetic energy (MD-*K*_e_) (Figure 6b,c), where a dramatic expansion into the vacuum region (Depth < 0) signifies the initiation of material ablation and plume ejection.

The temperature dynamics further highlight this non-equilibrium state. As quantified in Figure 6d, the electron temperature (*T*_e_) reaches a staggering peak of 3 × 10^5^ K at 0.2 ps, whereas the lattice temperature (*T*_a_) rises much more slowly, reaching equilibrium at approximately 31.5 ps. The spatiotemporal evolution of MD-*T*_e_ (Figure 6e) illustrates the extreme, localized electronic heating at the surface, which subsequently thermalizes with the lattice. As captured in MD-*T*_a_ (Figure 6f), this process establishes a steep atomic temperature gradient that decays from the surface into the interior. This rapidly developing thermal gradient is the fundamental cause of thermoelastic stress, while simultaneously driving the material’s thermal expansion into the vacuum (Depth < 0).

**Figure 7:**
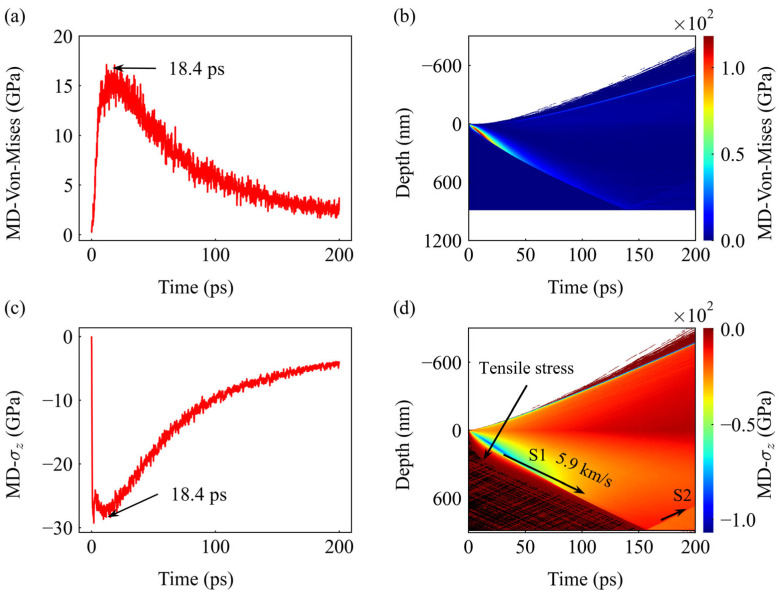
Stress response from TTM-MD simulation at 100 mW (1.97 J/cm^2^). (**a**) Time evolution of Von Mises stress at the surface center, peaking at ~17 GPa at 18.4 ps. (**b**) Spatiotemporal map of Von Mises stress propagating into the bulk copper. (**c**) Time evolution of axial stress (*σ_z_*), showing a peak compressive state of ~−30 GPa. (**d**) Spatiotemporal map of axial stress (*σ_z_*), identifying the primary compressive wave (S1) propagating at 5.9 km/s and the reflected tensile waves. The darker regions represent the tensile stress zones responsible for subsurface damage.

While the macroscopic TTM-FEM model serves as a numerical framework for predicting ablation depth, the TTM-MD simulation captures the intrinsic physical mechanisms. The intense temperature gradient launches a powerful thermoelastic shock wave into the material. As shown in Figure 7a, the von Mises equivalent stress peaks at approximately 17 GPa within 18.4 ps. The spatiotemporal map (Figure 7b) shows this stress wave propagating inwards, providing the mechanical foundation for subsequent material response.

As shown in Figure 7c, the axial stress (*σ_z_*) plays a dominant role in the mechanical response of the system. Under the action of the laser pulse, the axial stress increases rapidly, reaching a peak compressive state of approximately −30 GPa at 18.4 ps. In contrast, the peak von Mises equivalent stress at the same moment is only about 17 GPa (Figure 7a); this significant numerical discrepancy demonstrates that axial compression is the primary mechanical load within the material, directly driving the subsequent evolution of the shock wave.

Subsequently, the spatiotemporal map in Figure 7d further elucidates the dynamic propagation mechanism of this axial stress. The results reveal that the laser pulse triggers an intense primary compression wave (S1) propagating into the bulk at a supersonic velocity of 5.9 km/s, which matches well with the accepted speed of sound under high stress in copper [72]. Upon reaching the bottom boundary of the simulation domain, this wave reflects as a secondary wave (S2) and begins traveling back toward the surface. Notably, by the end of the 200 ps simulation period, the S2 wave has not yet reached the material surface, indicating that within the current observation scale, the deep reflected wave has not yet participated in or interfered with the initial surface ablation process.

At this power of 100 mW (1.97 J/cm^2^), these microscopic insights confirm that while the reflected S2 wave creates a complex mechanical environment within the deep regions of the target, its propagation lag prevents it from influencing the initial ablation. This demonstrates that at this energy level, the macroscopic ablation depth is primarily governed by surface evaporation kinetics, while internal mechanical wave propagation has not yet interfered with the plume ejection.

**Figure 8:**
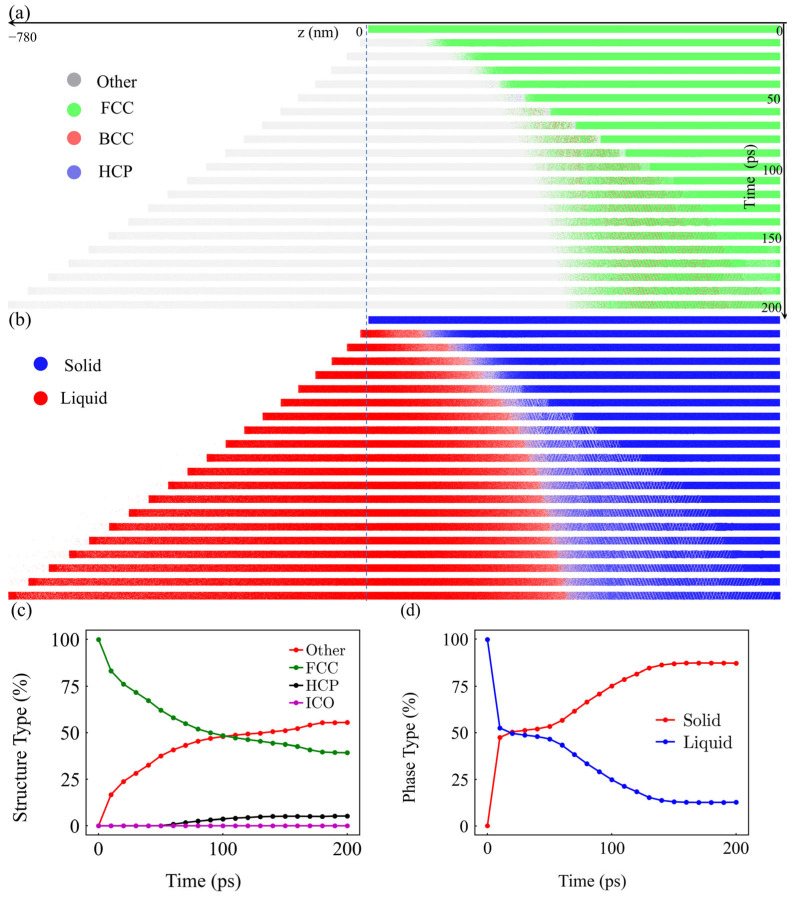
Atomic-scale structural evolution from TTM-MD simulation at 100 mW (1.97 J/cm^2^). (**a**) Spatiotemporal map of crystal structures (FCC, BCC, HCP, and Other). (**b**) Spatiotemporal map of solid/liquid phases (melting front). (**c**) Time evolution of structure type fractions. (**d**) Time evolution of solid/liquid phase fractions.

Following energy deposition and stress excitation, the material undergoes an irreversible structural response. Figure 8a depicts the spatiotemporal map of crystal structures, showing a compressive-stress-induced disordering front propagating inwards. The lattice undergoes significant phase transitions from FCC to Other, BCC, or HCP configurations due to the shock wave’s passage. Concurrently, the material undergoes transient melting (Figure 8b), tracking the evolution of the melting front and subsequent partial re-solidification.

Quantitative analysis in Figure 8c shows that the fraction of disordered atoms (“Other”) increases significantly, remaining as a permanent mechanical footprint in the remaining bulk. This is further supported by the phase type fractions in Figure 8d, illustrating the transition between solid and liquid phases. Even though the material removal depth is determined by thermal processes, the remaining bulk material retains high lattice distortion and structural damage.

### 3.4. Macroscopic Response via TTM-FEM Simulation

Building upon the microscopic stress and structural evolution elucidated via MD simulations, this section employs a multi-physics finite element method (FEM) model to investigate the femtosecond laser ablation of copper from a continuum perspective, aiming to bridge the vast spatio-temporal gap between atomistic dynamics and observable macroscopic outcomes. This framework achieves a self-consistent coupling of the Two-Temperature Model (TTM), solid mechanics, and the Arbitrary Lagrangian-Eulerian (ALE) moving mesh technique to simultaneously predict material removal, temperature field evolution, and stress dynamics.

**Figure 9:**
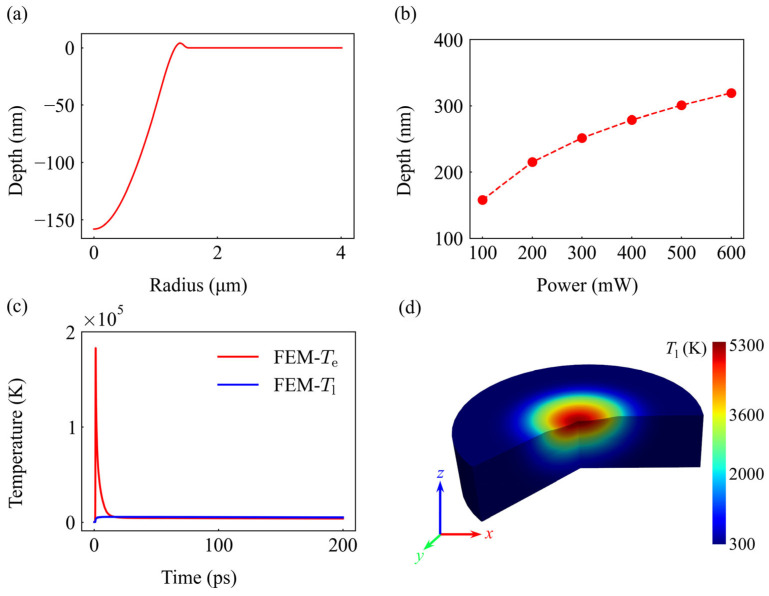
FEM simulation results of the thermo-ablation process. (**a**) Profile of the simulated ablation crater at 100 mW (1.97 J/cm^2^), showing a peak depth of ~160 nm. (**b**) Correlation between ablation depth and laser power, showing a non-linear increase in material removal. (**c**) Transient temperature profiles of the electron (*T*_e_) and lattice (*T*_l_) subsystems at the surface center at 100 mW (1.97 J/cm^2^), (**d**) 3D spatial map indicating the final crater location and thermal distribution at 100 mW (1.97 J/cm^2^).

Within this model, material removal is driven by the Hertz-Knudsen equation based on the enthalpy of vaporization, with the computational domain boundaries dynamically updated via the ALE technique. At a representative laser power of 100 mW, the simulation yields an ablation crater with a characteristic Gaussian-like profile and a peak central depth of approximately 160 nm, as illustrated in Figure 9a. Furthermore, the model indicates that as the incident power increases from 100 mW to 600 mW, the ablation depth exhibits a non-linear growth (Figure 9b). This trend aligns well with experimental observations, validating the model’s predictive fidelity in the low-to-medium power regime.

**Figure 10:**
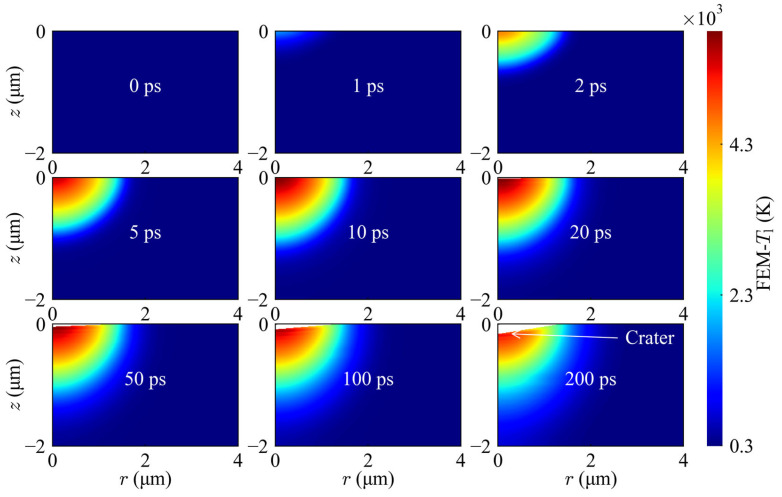
Spatiotemporal evolution maps of the lattice temperature (*T*_l_) at 100 mW (1.97 J/cm^2^).

The underlying thermodynamic journey at 100 mW (1.97 J/cm^2^) reveals a profound state of non-equilibrium. Upon laser pulse arrival, the electron temperature at the surface center skyrockets to approximately 1.8 × 10^5^ K within a picosecond (Figure 9c). Through electron-phonon coupling, energy is subsequently funneled to the lattice, causing a gradual rise in lattice temperature. The 3D spatial map confirms a maximum lattice temperature of 5300 K, precisely coinciding with the final crater location—effectively linking transient thermodynamic excitation to macroscopic material removal. The spatiotemporal odyssey in Figure 10 clearly illustrates the dynamic trajectory from localized energy absorption and peak lattice thermalization to subsequent thermal diffusion; simultaneously, it captures the complete process of incremental material removal driven by the advancing ablation front under sustained high temperatures.

**Figure 11:**
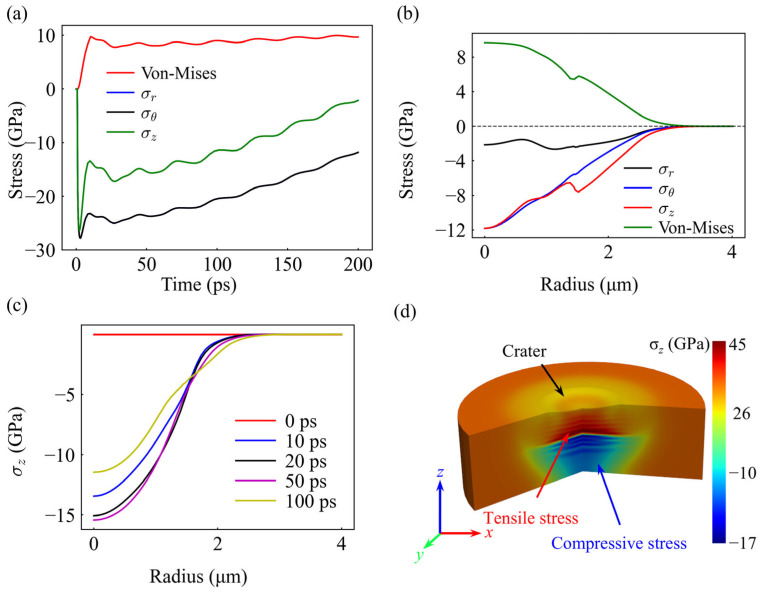
Stress evolution from FEM simulation at 100 mW (1.97 J/cm^2^). (**a**) Time evolution of stress components at the surface center. (**b**) Radial distribution of stress at 200 ps. (**c**) Radial distribution of axial stress (*σ_z_*) at different time intervals. (**d**) 3D cross-sectional distribution of axial stress at 200 ps, highlighting the subsurface tensile and compressive regions.

The intense thermal excitation simultaneously triggers a drastic mechanical response characterized by complex, multi-axial stress evolution. As depicted in Figure 11a, the intense laser-induced thermal excitation triggers a significant mechanical response at the surface center. Initially, the von Mises equivalent stress climbs rapidly and eventually stabilizes at a plateau of approximately 10 GPa, which supports the validity of the transient mechanical response in our simulation that leads to this state [73]; its subsequent wave-like oscillations vividly mark the internal evolution and relaxation of transient stresses, culminating in a persistent residual stress state. Regarding the specific stress components, the axial stress (*σ_z_*) reaches a compressive peak of approximately −28 GPa within the first few picoseconds—a direct result of the strong constraint from the cold material surrounding the irradiated volume. This peak magnitude significantly exceeds the radial (*σ_r_*) and tangential (*σ_θ_*) stress components, which exhibit nearly identical values. This finding is highly consistent with the previously discussed TTM-MD simulations regarding the dominance of axial stress, further confirming from a macroscopic perspective that axial compression serves as the core mechanical driver for material ablation.

Figure 11b illustrates the radial distribution characteristics of the stress, which exhibits a strong correlation with the laser energy profile. Due to the peak energy density at the center of the Gaussian beam, the stress concentration is most pronounced at the center (Radius = 0) and smoothly decays toward the periphery with increasing radial distance. This distribution pattern provides direct evidence that the local stress intensity is a spatial mapping of the incident laser energy flux.

Figure 11c further delineates the temporal evolution of the axial stress (*σ_z_*). Driven by laser-induced transient thermal expansion, the magnitude of compressive *σ_z_* intensifies during the initial stage, reaching its evolutionary peak between 20 and 50 ps. Subsequently, as thermal energy diffuses into the bulk and the material ablation process proceeds, the internal stress enters a relaxation phase, characterized by a gradual decrease in magnitude. This “intensification-then-relaxation” trajectory captures the complete dynamic history within the material, spanning from intense mechanical excitation to eventual energy dissipation.

**Figure 12:**
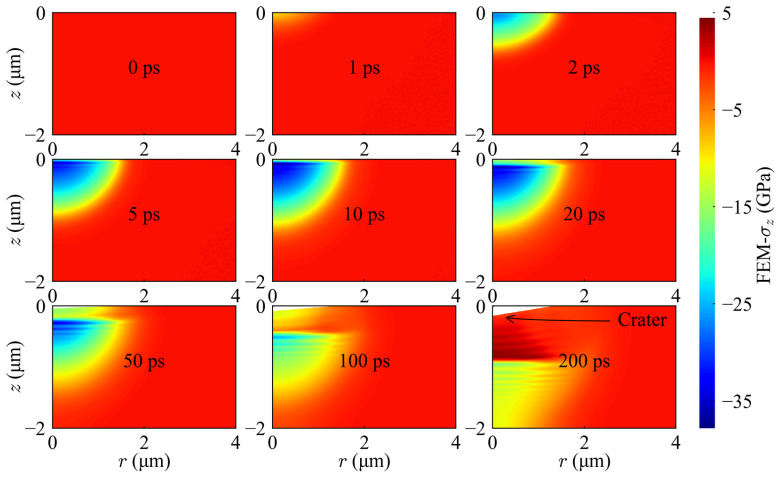
Spatiotemporal evolution maps of the axial stress (*σ_z_*) at 100 mW (1.97 J/cm^2^).

Crucially, the 3D cross-sectional view (Figure 11d) and the spatiotemporal evolution map (Figure 12) collectively reveal a unique mechanical evolution logic at 100 mW. As illustrated in Figure 12, during the critical ablation phase before 100 ps, the system interior is predominantly governed by compressive waves, with no early-stage tensile stress induced by acoustic reflections. This phenomenon provides robust mechanical evidence that, at the current power density, material removal does not originate from mechanical spallation but is instead dominated by surface evaporation kinetics.

Further observation of Figure 11d reveals a significant subsurface tensile stress layer (red region) at 200 ps, a point when the ablation process is largely completed. Based on the temporal evolution trends, it can be confirmed that this tensile stress is not a product of shock wave reflection, but rather forms during the cooling contraction and solidification phases following ablation. This finding carries profound physical significance: it demonstrates that at the 100 mW energy level, the macroscopic ablation depth is determined by thermodynamic evaporation, while the subsequent cooling process leaves a distinct mechanical footprint in the subsurface via thermal contraction. This late-stage residual tensile stress accounts for the lattice distortion and high dislocation density observed in experimental KAM maps, thereby establishing a self-consistent physical picture spanning from transient evaporative ablation to subsequent condensation-induced damage.

### 3.5. Comparative Analysis and Unified Ablation Mechanism

This study culminates in a systematic cross-scale comparison of results from experimental observations, MD simulations, and FEM simulations. This analysis validates the integrated framework and reveals a self-consistent physical picture of the femtosecond laser ablation of copper, bridging the gap between atomistic dynamics and macroscopic results.

**Figure 13:**
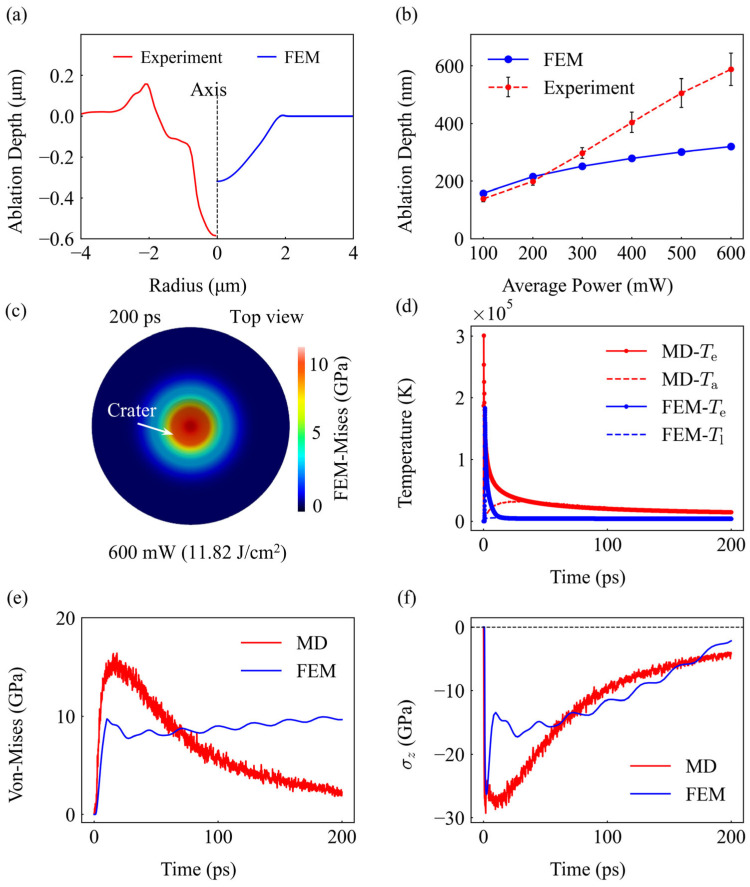
Cross-scale comparative analysis of experimental, MD, and FEM results. (**a**) Comparison of ablation crater profiles at 600 mW, highlighting the discrepancy in melt protrusions and peak depth. (**b**) Ablation depth as a function of average laser power, demonstrating the model’s validity below 300 mW and the divergence due to phase explosion at higher powers. (**c**) Top view of surface Mises stress at 200 ps (FEM, 600 mW), showing the residual stress distribution around the crater. (**d**) Comparison of electron (*T*_e_) and lattice/ion (*T*_l_/*T*_a_) temperature evolution at 100 mW. (**e**) Comparison of Von Mises stress evolution at 100 mW. (**f**) Comparison of axial stress (*σ_z_*) evolution at 100 mW, demonstrating identical dynamic trends across scales.

A comparison of the final ablation morphology reveals fundamental agreements and instructive discrepancies between the simulation and experimental results. At 600 mW, both the FEM profile and the experimental crater exhibit a consistent Gaussian-like geometry (Figure 13a). However, the experimental profile displays distinct melt protrusions at the rim, likely resulting from the splashing and rapid resolidification of molten material—a fluid dynamics phenomenon not currently accounted for in the solid-mechanics-based FEM framework.

Regarding the ablation depth (Figure 13b), the FEM prediction aligns excellently with experimental data in the low-to-medium power range (100–300 mW), where material removal is effectively governed by surface evaporation kinetics. At higher powers (>400 mW), the experimental depth increases more rapidly to ~600 nm, while the evaporation-based FEM model underestimates it at ~320 nm. This divergence underscores a mechanistic shift: at high energy densities, the ablation process transitions from simple surface evaporation to more efficient, thermally-dominated mechanisms such as phase explosion. Compared to the existing literature, some purely thermal models report a slightly lower deviation of approximately 5.69% [43]. However, it is noteworthy that such high precision is often achieved through technical processing rather than comprehensive physical modeling. For instance, several models artificially truncate the simulation or define the ablation depth once the material temperature reaches a specific threshold (e.g., 0.9*T_c_*), which lacks a rigorous thermo-mechanical basis. In contrast, our multi-scale framework avoids such non-physical shortcuts. By integrating a dedicated mechanical module, our model accounts for the dynamic stress–strain response and material deformation. While this increases the complexity of the calculation and leads to a slightly higher error margin (averaging 8.28–15.39% in the stable regime), it provides a more authentic and predictive representation of the physical ablation process, bridging the gap between microscopic electronic excitation and macroscopic mechanical failure.

The macroscopic FEM simulation successfully captures the final mechanical state of the target even where removal mechanisms differ. Figure 13c presents the top view of the surface von Mises stress at 200 ps (100 mW), revealing a significant ring of concentrated residual stress at the crater rim. This spatial distribution is in excellent agreement with the experimental KAM map (Figure 4c), which also identifies high stress concentrations at the crater edge. This correlation validates the FEM model’s accuracy in predicting the macroscopic thermo-mechanical signature left by the laser interaction.

A direct comparison between the microscopic (MD) and macroscopic (FEM) models at 100 mW provides further insight into the influence of spatial scale. As shown in Figure 13d, a notable discrepancy exists in the peak electron temperature (*T*_e_); the MD simulation predicts a peak value approximately 1.6 times higher than that of the FEM model. This difference is attributed to the inherent nature of the two frameworks: while the MD model captures localized, discrete energy states at the atomistic level, the FEM continuum approach averages the thermal response over finite mesh volumes, naturally leading to a damped peak temperature. Despite this, the lattice temperatures (*T*_l_ and *T*_a_) converge toward a similar thermalization state, confirming the consistency of energy transfer across scales.

In contrast to the temperature peaks, the mechanical responses in Figure 13e,f show remarkable numerical consistency. The von Mises and axial stress (*σ_z_*) magnitudes predicted by both models are highly comparable, reflecting a unified mechanical response to the thermal load. Most importantly, both models converge on an identical core physical logic: the axial stress exhibits a clear “intensification-then-relaxation” (increase-then-decrease) trajectory (Figure 13f). In both cases, the compressive stress surges to a maximum within the first few picoseconds due to constrained thermal expansion and subsequently relaxes as energy dissipates into the bulk. This cross-scale corroboration demonstrates that the transient mechanical evolution is a universal signature of the interaction, providing a validated predictive tool for optimizing the structural integrity of laser-processed surfaces.

## 4. Conclusions

In conclusion, this work delivers a comprehensive cross-scale investigation into the co-evolution of thermal and mechanical responses in femtosecond laser-irradiated copper using an integrated DFT, TTM-MD, and TTM-FEM framework. We have demonstrated that while surface evaporation kinetics serve as the primary driver for ablation depth in the low-to-medium power range (up to 300 mW), the laser-induced thermo-mechanical coupling dictates the final mechanical state of the target.

The framework was validated through a unique “dynamic logic” consistency across scales. While MD and FEM simulations differ in peak magnitudes due to spatial averaging, they converge on an identical “intensification-then-relaxation” stress trajectory. Crucially, at 100 mW, the research reveals that the high-magnitude compressive surge does not trigger early mechanical spallation; instead, the persistent tensile stress observed in the final state originates from thermal contraction during the cooling and solidification phase. This finding provides a direct physical explanation for the localized residual stress concentration observed in experimental KAM maps at the crater periphery. Furthermore, the significant divergence between the evaporation-based model and experimental results at higher powers (>400 mW) identifies a critical mechanistic transition toward the phase explosion regime. By bridging atomistic insights with macroscopic outcomes, this research establishes a high-fidelity predictive tool for precision laser processing, offering a robust foundation for optimizing both the geometrical accuracy and the structural integrity of ultrafast laser manufacturing. The model demonstrates superior physical fidelity compared to the literature by integrating mechanical modules, achieving a balance between predictive accuracy and mechanistic completeness.

To further enhance the predictive fidelity of this multiscale framework, future research will focus on the following refinements: In the macroscopic TTM-FEM model, phase explosion mechanisms will be integrated alongside hydrodynamic modules to account for Marangoni flow and recoil pressure-driven melt dynamics. By incorporating thermodynamic state-based removal criteria, the model will be able to capture the physical transition from surface evaporation to explosive volumetric ablation, thereby resolving the predictive divergence observed at higher powers. Additionally, for the microscopic TTM-MD simulations in LAMMPS, we aim to implement dynamically updated, temperature-dependent electron-phonon coupling constants (*G*) and surface reflectivity (*R*) to replace the current constant-value assumptions. These advancements will provide a more robust theoretical foundation for optimizing both geometrical accuracy and structural integrity in precision ultrafast laser manufacturing.

## Figures and Tables

**Table 1:** Summary of experimental parameters for femtosecond laser processing and microstructural characterization.

Category (Units)	Parameters	Specifications/Methods
Laser source	System model	FemtoLAB (Altechna R&D, Lithuania)
	Wavelength	1028 nm
	Pulse duration (*τ*)	190 fs
	Repetition rate (kHz)	100
	Beam quality (*M*^2^)	<1.2
Laser processing	Polarization	Linear polarization
	Focusing optic	High-precision objective lens (50×)
	Beam waist radius (*w*_0_)	0.9 μm
	Numerical Aperture (NA)	0.42
Imaging	Equipment	Tescan MIRA3LMH Field Emission SEM
Morphology	Equipment	Zygo NewView 9000 3D Optical Profiler
	Measurement principle	Coherence Scanning Interferometry
	Vertical resolution	<0.1 nm
EBSD	Equipment	Tescan CLARA Field Emission SEM
	EBSD detector	High-speed low-noise CMOS camera
	CMOS detector	1244 × 1024 pixels
	Acceleration voltage	20 kV
	Step size	100 nm
	Angular resolution	Better than 0.05°
	KAM calculation	1st nearest neighbors, 5° threshold

**Table 2:** Key physical parameters for TTM-MD simulations [55,62,63,64,65].

Parameter (Unit)	Symbol	Value
Optical penetration depth (nm)	*δ*	44
Ballistic electron transport distance (nm)	*δ*_b_	58
Electron-phonon coupling constant (W/K/m^−3^)	*G*	1 × 10^17^
Reflectivity	*R*	0.95
Electron thermal conductivity (W/m/K)	*κ*_e_	*D*_e_ * × C*_e_
Absorbed Fluence (J/cm^2^)	*F*	1.97 (100 mW)
Electron diffusion coefficient (cm^2^/s)	*D*_e_	2

**Table 3:** Key physical parameters for TTM-FEM simulations [20,64,67,68,69,70,71].

Parameter (Unit)	Symbol	Value
Density of lattice (kg/m^3^)	*ρ*_l_	8935
Lattice thermal conductivity (W/m/K)	*κ*_l_	386
Enthalpy of vaporization (J/kg)	*L*_v_	2.5 × 10^6^
Melting point (K)	*T*_m_	1357
Boiling point (K)	*T*_v_	2836
Electron diffusion coefficient (cm^2^/s)	*D*_e_	2
Electron thermal conductivity (W/m/K)	*κ*_e_	*D*_e_ × *C*_e_
Young’s modulus (GPa)	*E*	116.7
Poisson’s ratio	*ν*	0.34
Linear thermal expansion coefficient (K^−1^)	*α*	16.5 × 10^−6^
Electron-phonon coupling constant (W/K/m^−3^)	*G*	1 × 10^17^
Solver		MUMPS
Mesh strategy		Mapped distribution
Element type		4-node quadrilateral (Square)
Element size (nm)		25
Number of degrees of freedom (DOFs)		284,807
Time-stepping scheme		Generalized α
Initial time step (fs)		1
Step control method		Strict
Relative tolerance		1 × 10^−4^

## Data Availability

The original contributions presented in this study are included in the article/Supplementary Materials. Further inquiries can be directed to the corresponding authors.

## Supplementary Materials

The following supporting information can be downloaded at: https://www.mdpi.com/article/10.3390/ma19071391/s1, Figure S1: Mesh independence validation; Figure S2: Time-step independence validation.

## References

[1] Orazi L., Romoli L., Schmidt M., Li L. (2021). Ultrafast laser manufacturing: From physics to industrial applications. CIRP Ann..

[2] Wang S., Yang J., Deng G., Zhou S. (2024). Femtosecond Laser Direct Writing of Flexible Electronic Devices: A Mini Review. Materials.

[3] Gao L., Zhang Q., Gu M. (2024). Femtosecond laser micro/nano processing: From fundamental to applications. Int. J. Extreme Manuf..

[4] Li D. (2023). Femtosecond pulsed laser technology and applications. Theor. Nat. Sci..

[5] Filipek K., Pisklak A., Behrendt H., Głód M., Węgrzynek M. (2024). Ultrashort pulse lasers in medicine, current applications and prospects for future advances. Med. Sci..

[6] Guo B., Sun J., Hua Y., Zhan N., Jia J., Chu K. (2020). Femtosecond Laser Micro/Nano-manufacturing: Theories, Measurements, Methods, and Applications. Nanomanuf. Metrol..

[7] Sugioka K., Cheng Y. (2014). Femtosecond laser three-dimensional micro- and nanofabrication. Appl. Phys. Rev..

[8] Shin S. (2024). Review of high-precision femtosecond laser materials processing for fabricating microstructures: Effects of laser parameters on processing quality, ablation efficiency, and microhole shape. J. Laser Appl..

[9] Shin S., Hur J.-G., Park J.K., Kim D.-H. (2021). Thermal damage free material processing using femtosecond laser pulses for fabricating fine metal masks: Influences of laser fluence and pulse repetition rate on processing quality. Opt. Laser Technol..

[10] Fuentes-Edfuf Y., Garcia-Lechuga M., Solis J., Siegel J. (2022). Ultrafast Electron Dynamics and Optical Interference Tomography of Laser Excited Steel. Laser Photonics Rev..

[11] Pompili R., Anania M.P., Bisesto F., Botton M., Castellano M., Chiadroni E., Cianchi A., Curcio A., Ferrario M., Galletti M. (2016). Femtosecond dynamics of energetic electrons in high intensity laser-matter interactions. Sci. Rep..

[12] Rethfeld B., Sokolowski-Tinten K., von der Linde D., Anisimov S.I. (2004). Timescales in the response of materials to femtosecond laser excitation. Appl. Phys. A.

[13] Shugaev M.V., Wu C., Armbruster O., Naghilou A., Brouwer N., Ivanov D.S., Derrien T.J.Y., Bulgakova N.M., Kautek W., Rethfeld B. (2016). Fundamentals of ultrafast laser–material interaction. MRS Bull..

[14] Li X., Guan Y. (2020). Theoretical fundamentals of short pulse laser–metal interaction: A review. Nanotechnol. Precis. Eng..

[15] Guo B., Sun J., Lu Y., Jiang L. (2019). Ultrafast dynamics observation during femtosecond laser-material interaction. Int. J. Extreme Manuf..

[16] Pompili R., Anania M.P., Bisesto F., Botton M., Chiadroni E., Cianchi A., Curcio A., Ferrario M., Galletti M., Henis Z. (2018). Ultrafast evolution of electric fields from high-intensity laser-matter interactions. Sci. Rep..

[17] Li Y.Y., Gao B., Ma C.Y., Wu G., Huo J.Y., Han Y., Wageh S., Al-Hartomy O.A., Al-Sehemi A.G., Liu L. (2023). Generation of High-Peak-Power Femtosecond Pulses in Mamyshev Oscillators: Recent Advances and Future Challenges. Laser Photonics Rev..

[18] Miloshevsky G. (2022). Ultrafast laser matter interactions: Modeling approaches, challenges, and prospects. Modell. Simul. Mater. Sci. Eng..

[19] Parris G., Goel S., Nguyen D.T., Buckeridge J., Zhou X. (2022). A critical review of the developments in molecular dynamics simulations to study femtosecond laser ablation. Mater. Today Proc..

[20] Zhan N., Guo B., Jiang L., Zhang T., Chen M., Lin G. (2023). Multiphysics modeling of femtosecond laser–copper interaction: From electron dynamics to plasma eruption. Phys. Fluids.

[21] Capone M., Romanelli M., Castaldo D., Parolin G., Bello A., Gil G., Vanzan M. (2024). A Vision for the Future of Multiscale Modeling. ACS Phys. Chem. Au.

[22] Fletcher A.G., Osborne J.M. (2022). Seven challenges in the multiscale modeling of multicellular tissues. WIREs Mech. Dis..

[23] Yao Y.N., Harabin P., Behandish M., Battiato I. (2023). Non-intrusive hybrid scheme for multiscale heat transfer: Thermal runaway in a battery pack. J. Comput. Sci..

[24] Feng T., Chen G., Han H., Qiao J. (2021). Femtosecond-Laser-Ablation Dynamics in Silicon Revealed by Transient Reflectivity Change. Micromachines.

[25] Kudryashov S.I., Ionin A.A. (2016). Multi-scale fluence-dependent dynamics of front-side femtosecond laser heating, melting and ablation of thin supported aluminum film. Int. J. Heat Mass Transf..

[26] Zhang N., Zhu X., Yang J., Wang X., Wang M. (2007). Time-resolved shadowgraphs of material ejection in intense femtosecond laser ablation of aluminum. Phys. Rev. Lett..

[27] Wu J., Zhou E., Huang A., Zhang H., Hu M., Qin G. (2024). Deep-potential enabled multiscale simulation of gallium nitride devices on boron arsenide cooling substrates. Nat. Commun..

[28] Song S., Lu Q., Zhang P., Yan H., Shi H., Yu Z., Sun T., Luo Z., Tian Y. (2023). A critical review on the simulation of ultra-short pulse laser-metal interactions based on a two-temperature model (TTM). Opt. Laser Technol..

[29] Grigoryeva M.S., Kutlubulatova I.A., Lukashenko S.Y., Fronya A.A., Ivanov D.S., Kanavin A.P., Timoshenko V.Y., Zavestovskaya I.N. (2023). Modeling of Short-Pulse Laser Interactions with Monolithic and Porous Silicon Targets with an Atomistic-Continuum Approach. Nanomaterials.

[30] Wu C., Zhigilei L.V. (2013). Microscopic mechanisms of laser spallation and ablation of metal targets from large-scale molecular dynamics simulations. Appl. Phys. A.

[31] Ji P., Zhang Y. (2017). Multiscale modeling of femtosecond laser irradiation on a copper film with electron thermal conductivity from ab initio calculation. Numer. Heat Transf. Part A Appl..

[32] Zhou Y., Wu D., Luo G., Hu Y., Qin Y. (2022). Efficient modeling of metal ablation irradiated by femtosecond laser via simplified two-temperature model coupling molecular dynamics. J. Manuf. Process..

[33] Lian Y., Jiang L., Sun J., Lin G., Liang M. (2024). Atomistic insight on temperature-dependent laser induced ultrafast thermomechanical response in aluminum film. Int. J. Heat Mass Transf..

[34] Ganesan H., Sandfeld S. (2025). Capturing thin-film microstructure contributions during ultrafast laser-metal interactions using atomistic simulations. Mater. Des..

[35] Benhayoun O., Garcia M.E. (2026). Multiscale Numerical Modelling of Ultrafast Laser-Matter Interactions: Maxwell Two Temperature Model Molecular Dynamics (M-TTM-MD). arXiv.

[36] Rethfeld B., Ivanov D.S., Garcia M.E., Anisimov S.I. (2017). Modelling ultrafast laser ablation. J. Phys. D Appl. Phys..

[37] Omeñaca L., Olaizola S.M., Rodríguez A., Gomez-Aranzadi M., Ayerdi I., Castaño E. (2025). Experimental findings and 2 dimensional two-temperature model in the multi-pulse ultrafast laser ablation on stainless steel considering the incubation factor. Opt. Laser Technol..

[38] Liu D., Chen C., Man B., Meng X., Sun Y., Li F. (2015). Experimental investigation and 3D-simulation of the ablated morphology of titanium surface using femtosecond laser pulses. Eur. Phys. J. Appl. Phys..

[39] Akarapu R., Li B.Q., Segall A. (2004). A thermal stress and failure model for laser cutting and forming operations. J. Fail. Anal. Prev..

[40] Tsibidis G.D., Stratakis E., Aifantis K.E. (2012). Thermoplastic deformation of silicon surfaces induced by ultrashort pulsed lasers in submelting conditions. J. Appl. Phys..

[41] Redka D., Vollmann J., Winter J., Schmidt M., Minár J., Huber H.P., Schmid P. (2025). Improving FEM-based solid mechanics simulations for ultrashort pulse laser ablation by integrating an equation of state and material separation. Int. J. Heat Mass Transf..

[42] Liu Y., Claus S., Kerfriden P., Chen J., Zhong P., Dolbow J.E. (2023). Model-based simulations of pulsed laser ablation using an embedded finite element method. Int. J. Heat Mass Transf..

[43] Xu Z., Zhao N., Zhang C., Cai S., Wang K., Wang K., Li J., Zhang Q., Yan X., Zhu K. (2024). Accurate two-dimensional simulation model and experimental demonstration in ultraviolet picosecond laser scribing ablation. Opt. Express.

[44] Calogero G., Raciti D., Acosta-Alba P., Cristiano F., Deretzis I., Fisicaro G., Huet K., Kerdilès S., Sciuto A., La Magna A. (2022). Multiscale modeling of ultrafast melting phenomena. npj Comput. Mater..

[45] Zhao J., Han X., Dong F., Liu S. (2025). Innovative multiscale simulation with experimental validation of ultrafast laser processing in silicon carbide (4H-SiC). J. Manuf. Process..

[46] Light Conversion PHAROS: Automated High-Power Femtosecond Laser System Technical Specifications. https://lightcon.com/products/pharos-femtosecond-lasers/.

[47] Workshop of Photonics FemtoLAB: Femtosecond Laser Micromachining System Product Portfolio. https://wophotonics.com/products/femtolab/.

[48] Tescan Orsay Holding, a.s. TESCAN MIRA: Analytical Field Emission Scanning Electron Microscope. https://tescan.com/product-portfolio/sem/mira.

[49] Zygo Corporation NewView™ 9000 3D Optical Profiler Datasheet and Specifications. https://www.zygo.com/products/metrology-systems/3d-optical-profilers/newview-9000.

[50] Tescan Orsay Holding, a.s. TESCAN CLARA: Ultra-High Resolution Scanning Electron Microscope for Materials Science. https://tescan.com/product-portfolio/sem/clara.

[51] Lin Z., Zhigilei L.V., Celli V. (2008). Electron-phonon coupling and electron heat capacity of metals under conditions of strong electron-phonon nonequilibrium. Phys. Rev. B.

[52] Giannozzi P., Baroni S., Bonini N., Calandra M., Car R., Cavazzoni C., Ceresoli D., Chiarotti G.L., Cococcioni M., Dabo I. (2009). Quantum ESPRESSO: A modular and open-source software project for quantum simulations of materials. J. Phys. Condens. Matter.

[53] Pedroza L.S., da Silva A.J.R., Capelle K. (2009). Gradient-dependent density functionals of the Perdew-Burke-Ernzerhof type for atoms, molecules, and solids. Phys. Rev. B.

[54] Zeng Q., Chen B., Zhang S., Kang D., Wang H., Yu X., Dai J. (2023). Full-scale ab initio simulations of laser-driven atomistic dynamics. npj Comput. Mater..

[55] Zarkadoula E., Shinohara Y., Egami T. (2022). X-ray free-electron laser heating of water at picosecond time scale. Phys. Rev. Res..

[56] Thompson A.P., Aktulga H.M., Berger R., Bolintineanu D.S., Brown W.M., Crozier P.S., in ‘t Veld P.J., Kohlmeyer A., Moore S.G., Nguyen T.D. (2022). LAMMPS—A flexible simulation tool for particle-based materials modeling at the atomic, meso, and continuum scales. Comput. Phys. Commun..

[57] Chen J.K., Tzou D.Y., Beraun J.E. (2025). A semiclassical two-temperature model for ultrafast laser heating. Int. J. Heat Mass Transf..

[58] Norman G.E., Starikov S.V., Stegailov V.V., Saitov I.M., Zhilyaev P.A. (2013). Atomistic Modeling of Warm Dense Matter in the Two-Temperature State. Contrib. Plasma Phys..

[59] Stukowski A. (2010). Visualization and analysis of atomistic simulation data with OVITO–the Open Visualization Tool. Modell. Simul. Mater. Sci. Eng..

[60] Honeycutt J.D., Andersen H.C. (1987). Molecular dynamics study of melting and freezing of small Lennard-Jones clusters. J. Phys. Chem..

[61] Larsen P.M., Schmidt S., Schiøtz J. (2016). Robust structural identification via polyhedral template matching. Modell. Simul. Mater. Sci. Eng..

[62] Mattern M., Kukreja L.M., Ostendorf A. (2023). Temperature-Dependent Reflectance of Copper with Different Surface Conditions Measured at 1064 nm. J. Mater. Eng. Perform..

[63] Miyasaka Y., Hashida M., Nishii T., Inoue S., Sakabe S. (2015). Derivation of effective penetration depth of femtosecond laser pulses in metal from ablation rate dependence on laser fluence, incidence angle, and polarization. Appl. Phys. Lett..

[64] Vanwersch P., Nagarajan B., Bael A.V., Castagne S. (2023). Femtosecond Laser Ablation Modeling of Tilted Microfeatures on Copper Surfaces. J. Laser Micro/Nanoeng..

[65] Toulemonde M., Dufour C., Meftah A., Paumier E. (2000). Transient thermal processes in heavy ion irradiation of crystalline inorganic insulators. Nucl. Instrum. Methods Phys. Res. B.

[66] Nikolidakis E., Antoniadis A. (2019). FEM modeling simulation of laser engraving. Int. J. Adv. Manuf. Technol..

[67] Abdelmalek A., Bedrane Z., Amara E.-H., Sotillo B., Bharadwaj V., Ramponi R., Eaton S.M. (2018). Ablation of Copper Metal Films by Femtosecond Laser Multipulse Irradiation. Appl. Sci..

[68] Zhan N., Jiang L., Zhang T., Lian Y., Guo B. (2024). Lagrangian perspective on the expansion dynamics and shielding effect of femtosecond laser-induced copper plasma plumes. Phys. Fluids.

[69] Zhu H., Zhang Z., Zhou J., Xu K., Zhao D., Tangwarodomnukun V. (2021). A computational study of heat transfer and material removal in picosecond laser micro-grooving of copper. Opt. Laser Technol..

[70] Paszyński M. (2013). Minimizing the memory usage with parallel out-of-core multi-frontal direct solver. Comput. Assist. Methods Eng. Sci..

[71] Zdunek A. (2021). Tests with FALKSOL A massively parallel multi-level domain decomposing direct solver. Comput. Math. Appl..

[72] Hawreliak J.A., Winey J.M., Toyoda Y., Zimmerman K., Gupta Y.M. (2024). Sound speed determination in copper shock compressed to 190 GPa. J. Appl. Phys..

[73] Amer M.S., El-Ashry M.A., Dosser L.R., Hix K.E., Maguire J.F., Irwin B. (2005). Femtosecond versus nanosecond laser machining: Comparison of induced stresses and structural changes in silicon wafers. Appl. Surf. Sci..

