# Supplementary material for: Multi-Scale Modeling and Experimental Validation of Thermo-Mechanical Responses in Femtosecond Laser Micromachining of Copper
Source: Materials (Basel). 2026 Mar 31;19(7):1391. doi: 10.3390/ma19071391 (PMC13074060; doi:10.3390/ma19071391)
Supplement: Supplementary file 1 [file materials-19-01391-s001.zip › materials-4205596-supplementary.pdf]

Supplementary Information for "Multi-scale Modeling and Experimental Validation of Thermo-Mechanical Responses in Femtosecond Laser Micromachining of Copper"

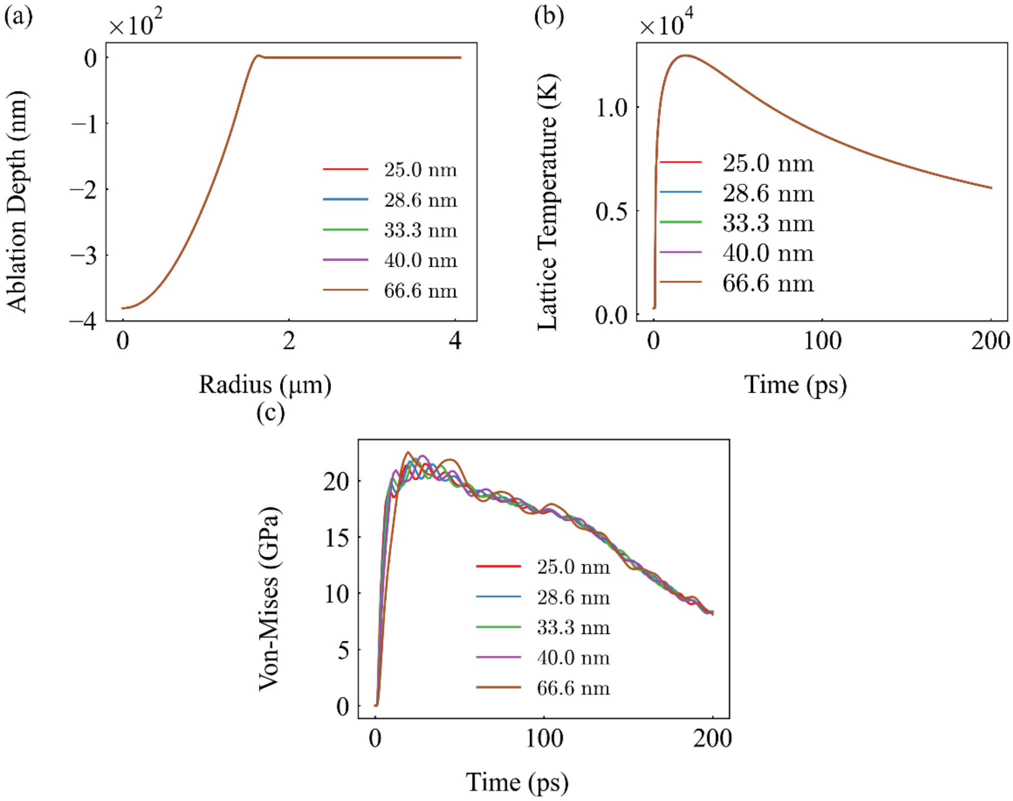

Figure S1. Mesh independence validation. Profiles of (a) ablation depth, (b) lattice temperature, and (c) Von-Mises stress calculated with different element sizes (66.6 nm, 40.0 nm, and 25.0 nm) at a laser power of 600 mW. The results demonstrate that spatial convergence is achieved at an element size of 25.0 nm.

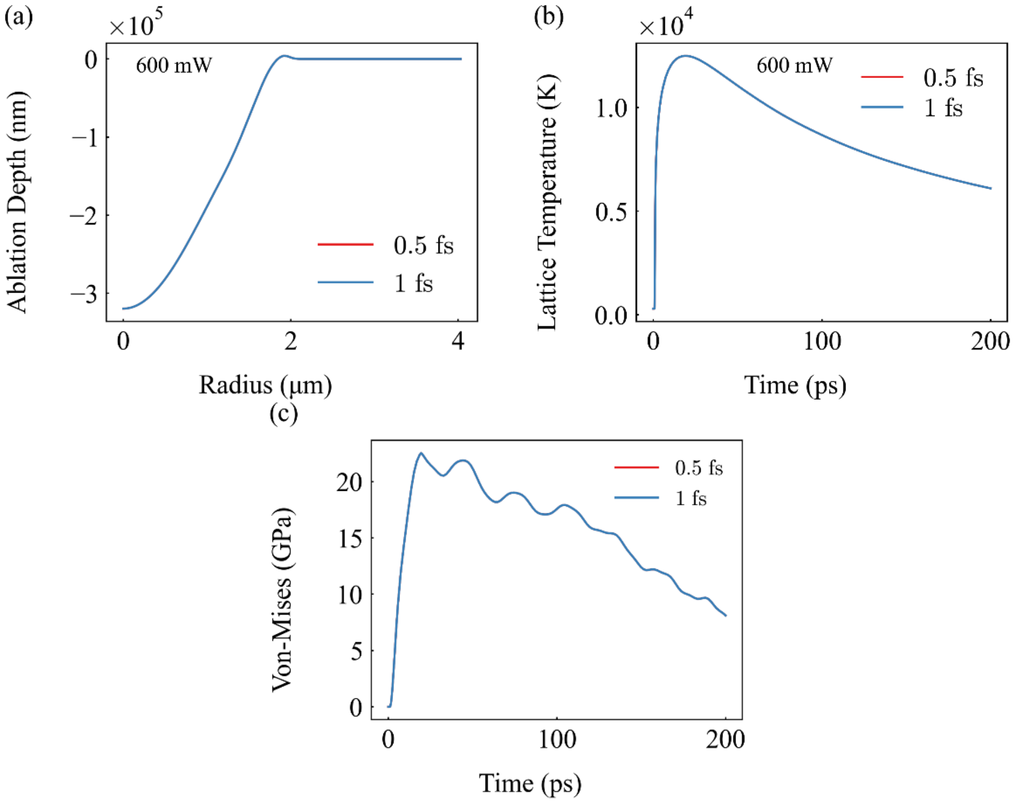

Figure S2. Time-step independence validation. Comparison of (a) ablation depth, (b) lattice temperature, and (c) Von-Mises stress using initial time steps of 1 fs (0.001 ps) and 0.5 fs at 600 mW. The identical overlapping curves confirm that the 1fs time step provides sufficient temporal resolution for the transient multiphysics simulation.
